# The Influence of Ink Chemistry on the Microstructure Evolution and GHz RF Response of Printed Ag Transmission Lines

**DOI:** 10.3390/ma17081756

**Published:** 2024-04-11

**Authors:** Jason M. Summers, Shambhavi Sakri, Nishako Chakma, Hung Luyen, Andres Bujanda, Thomas Parker, Harvey Tsang, Nigel D. Shepherd

**Affiliations:** 1 Department of Materials Science and Engineering, University of North Texas, Denton, TX 76207, USA; 2 Department of Electrical Engineering, University of North Texas, Denton, TX 76207, USA; nishakochakma@my.unt.edu (N.C.);; 3 DEVCOM Army Research Laboratory, Aberdeen Proving Ground, Aberdeen, MD 21005, USAthomas.c.parker84.civ@army.mil (T.P.);

**Keywords:** additive manufacturing, 3D printing, direct write, direct ink writing, ink chemistry, microstructure evolution, RF response, transmission line

## Abstract

High-frequency transmission is limited to the skin depth in metals. Because poor conductivity cannot be compensated for by increasing the conductor thickness as with DC, optimal transport properties are prerequisites for radio frequency (RF) use. Structural and chemical analyses of transmission lines printed using a traditional ink consisting of Ag nanoflakes in a dispersing phase revealed that optimized thermal treatments yielded thorough burnout of the binder, significant grain growth, elimination of the pore volume, and electrical responses that were comparable to values obtained for thermally evaporated, fully dense Ag controls. Specifically, a low DC resistivity of 2.3 μΩ·cm (1.4× bulk Ag) and RF transmission coefficients of 0.87 and 0.75 at 5 GHz and 10 GHz, respectively, were measured in the nanoflake Ag prints. Conversely, in transmission lines printed from a metal-organic decomposition ink, residual chemical contamination impeded diffusion and densification, yielding greater porosity, small grains that are pinned, and a degraded RF response. Reasonably good porosity approximations were obtained from a model based on percolation theory. The results indicate that contaminants at interfaces and pore surfaces impede diffusion, pore elimination, and full densification, and further, alter carrier dynamics and degrade RF response.

## 1. Introduction

Radio frequency (RF) and high-frequency circuits are the foundation of wireless communication technologies and enable a wide variety of applications, including wireless connectivity, mobile communications, the Internet of Things, vehicular radar, implanted devices, and wearable electronics. As the proliferation of RF expands, additive manufacturing (AM) methods such as direct-write (DW) 3D printing that enable innovative designs and accommodate rapid prototyping are being extensively investigated for adding functionality, packaging, and miniaturization of passive RF components that cannot be achieved with planar structures. For example, in recent years, flexible hybrid electronics (FHE) and 2D/3D architectures have progressed substantially thanks to innovations in additive manufacturing processing technologies [1,2].

Among direct write methods, extrusion-based direct write microdispensing (DWM) additive manufacturing technology is precise, capable of printing metals and dielectrics with complex shapes in the same platform [3], and covers dimensions ranging from microns to centimeters, thereby bridging the gap between micro- and macro-manufacturing processes [4,5,6,7], and its potential for printing 3D circuits has been demonstrated [8]. The CAD-based nature of DW systems such as those patented by nScrypt^TM^ (Orlando, FL, USA) brings several advantages associated with AM, such as reduced material waste, lower cost, rapid prototyping, reduced manufacturing time, complex and conformal geometry printing, and much more [3,9]. Production of several passive components has been demonstrated in the last decade by DW and other associated extrusion-based printing technologies [6,8,10,11,12]. However, a central factor limiting the widespread commercialization of printed electronics is poor performance due to inadequate materials quality and processing, and the core knowledge gap lies in how to achieve bulk properties in printed materials. In the context of RF, the goal of many researchers is to produce RF passives with equivalent (or comparable) microstructures, macrostructures, properties, and performance to their traditionally manufactured counterparts. Thus, substantial research remains to realize the full potential of additive manufacturing for producing high-performance RF components, such as transmission lines, interconnects, antennas, switches, and lumped elements for filters and resonators.

For interconnects and transmission lines, specifically, Ag is an excellent candidate because it has the highest electrical and thermal conductivity among all metals. Ag suspension inks based on nanoparticles, nanowires, or nanoflakes [13,14] are common since the large surface-to-volume ratios of nanoparticles significantly reduce sintering temperatures. The dispersing phase is typically some combination of polymers and solvents. An alternative to nanoparticle-based inks is reactive inks where thermally driven metal-organic decomposition (MOD) of a liquid precursor yields Ag precipitation from solution [15,16]. With the exception of [16], where Walker and Lewis reported that the DC conductivity of their low-viscosity reactive Ag ink was equivalent to bulk, the overwhelming majority of published values have been lower. For instance, Kim et al. [17] showed that the electrical conductivity of their 20 nm Ag nanoparticle ink was dependent on solvent composition, and they reported a resistivity of 3.5 μΩ·cm after 300 °C heat treatment, which is 2.2 times the 1.6 μΩ·cm for bulk Ag. Williams et al. [18] reported a high resistivity of 69 μΩ·cm using an Ag nanowire-based ink after heating to 80 °C. Copper inks have also been studied, and Sheng et al. [15] reported a conductivity of 3 × 10^7^ (Ω·m)^−1^ for their reactive Cu ink sintered at 250 °C, which is only ~half of the bulk Cu value of 5.96 × 10^7^ (Ω·m)^−1^.

Although these reported conductivities are adequate for DC applications and can be improved by increasing print thickness, RF transmission is limited to the skin depth. Thus, optimal structure and properties are prerequisites for minimizing attenuation losses and obtaining optimal performance at high frequencies. Because the mobility of charge carriers, defects, degree of crystallinity, etc. all contribute to frequency response, further investigation is required to understand the correlations among ink properties, post-print microstructural and chemical evolution from heat treatment, and electrical properties if bulk Ag properties and high-frequency performance are to be achieved. Given that the reduction of free interfacial energy (surface energy) is a fundamental driving force for densification and that grain boundaries are sinks for vacancies in sintering, the presence of the dispersing phase, its chemical composition, as well as the grain size distribution and shape are all important for microstructural evolution and resultant electrical properties. Thus, to obtain insight into the role of free interfacial energy, particle size, and shape in sintering and densification, a medium viscosity suspension containing 85 wt% of nanoflakes [14] with a D90 of 500 nm and an aspect ratio of 10 was compared with a low-viscosity reactive ink that included 36 wt% of less than 100 nm spherical nanoparticles [19]. The nanoflake ink specification sheet reports a room temperature viscosity of 25,000 cP at 10 Hz and a resistivity of 15.8 μΩ·cm after curing at 140 °C for 30 min, and this value improved to 6.2 μΩ·cm after curing at 225 °C for 5 min. The resistivity of the medium viscosity 2000 cP nanoparticle-reactive ink was not stated, although cure conditions of 100 °C for 20 min were recommended. This study used electrical characterization, scanning electron microscopy (SEM), X-ray diffraction (XRD), and X-ray photoelectron spectroscopy (XPS) to detail the relationship among thermally driven microstructural evolution, resistivity, and RF loss in two printed Ag inks for potential applications as flexible high-frequency transmission lines and interconnects. The results from this study show that with correct ink chemistry selection and optimal post-print heat treatment, the RF performance of printed Ag can be as good as evaporated equivalents.

## 2. Materials and Methods

### 2.1. Materials and Direct Write Setup

Commercially available Metalon HPS-FG77 nanoflake suspension and EI-610 nanoparticle-reactive Ag inks abbreviated as NF and NPR, respectively, were procured from their respective manufacturers, Novecentrix (Austin, TX, USA) and ElectronInks (Austin, TX, USA) [14,19], and printed without modification using a customized nScrypt 3Dn tabletop tool [3]. Samples were printed on glass slides, Si wafers, and DuPont Kapton substrates that were precleaned with acetone and methanol. Four-wire geometries were printed on glass slides for resistivity measurements. Samples that were prepared for XPS and XRD were printed on conductive Si wafers to eliminate potential charging in the XPS spectrometer, and RF transmission lines were printed on DuPont Kapton substrates because we are using this material for our work in flexible RF components. The following printing parameters were used: 75/125 μm nozzle size, print speed of 5 mm/s, and air pressure of 5–15 psi. The layer thickness was set at less than 10 μm for DC measurements and 2.5 μm for all coplanar waveguide samples.

### 2.2. Post-Print Thermal Treatment

Specimens were cured per the manufacturer’s recommendation and compared with samples subjected to heating at 225 °C and 350 °C with 30-min dwell times, the latter being the maximum temperature that the flexible DuPont Kapton polyimide substrates could accommodate before warping. The samples were heat treated in air using a traditional hotplate ramped from ambient to the dwell temperature at 12.5 °C/min. A K-type thermal couple was used to monitor the surface temperature of the print. Upon completion of the curing dwell time, the samples were immediately removed from the substrate heater to air cool.

### 2.3. Characterization of Electrical Properties, Microstructural, and Chemical Evolution

DC conductivity was obtained from four-wire measurements using samples printed on glass and a LabView-controlled Keithley 2420 source-measure unit (Cleveland, OH, USA) by sweeping current from −100 mA to 100 mA and measuring the voltage. The separation between the voltage measure pads (*L*) was 1 cm, with 2 cm between the current source connections. Average resistance was calculated using Ohms law, and the electrical resistivity (*ρ*) in μΩ·cm was obtained using Equation (1):

(1) $$\rho =\frac{RA}{L}$$

(2) $$\rho =\frac{1}{\sigma }$$

where *R* is the measured resistance, *A* is the cross-sectional area of the print, and *L* is its length. Equation (2) was used to convert from resistivity to conductivity (σ). The thickness of the printed samples was measured using a Veeco DEKTAK 150 stylus profilometer (Plainview, NY, USA), and the average trace widths were determined using optical microscopy. Using these measurements, the resistivity for each ink and thermal treatment condition was calculated from an average of at least three samples.

The microstructural changes resulting from the previously outlined thermal treatments were analyzed with SEM and XRD using a FEI Nova NanoSEM 230 instrument (Hillsboro, OR, USA) and Bruker D8 Discover spectrometer (Billerica, MA, USA), respectively. The latter employed a Gobel mirror yielding a parallel X-ray beam with both the K α1 and K α2 lines (1.5406 Å and 1.5444 Å) of a sealed Cu X-ray source and a 1-D Si strip detector with an effective step size of 0.0069 degrees. Crystallite size was obtained from Rietveld fitting of the XRD spectra using GSASII scattering analysis software (version 4955), from which the March-Dollase parameter was obtained and used to calculate the percentage orientation. MIPAR (version 4.4.0) image analysis software was used to estimate the porosity of the prints from SEM imaging. A PHI Versa Probe II XPS spectrometer (Chanhassen, MN, USA) operating in high-resolution mode with a 24 eV pass energy and 0.1 eV step size was used for chemical analysis. The adventitious C1s peak position (284.8 eV) was used to calibrate the energy axis. The surfaces of samples were sputter-cleaned with Ar^+^ at 3 kV prior to measurement.

### 2.4. RF Measurement Setup

To understand the correlation between microstructure and frequency response, RF analyses were performed in the 1–12 GHz range on the heat-treated NF and NPR samples with the highest DC conductivity using coplanar waveguide (CPW) structures. CST Microwave Studio was used to model the physical structure of the SMA connectors and coplanar waveguide (CPW) line in full-wave electromagnetic simulations. Experimental conductivity and dissipation factors were used to calculate the corresponding ohmic and dielectric losses of the Ag conductor and dielectric substrate. The SMA connectors were modeled with brass and polytetrafluoroethylene. Full-wave simulations were performed using CST Studio to derive numerical solutions for the electric and magnetic fields everywhere in the computational domain, and, in turn, that information was used to determine the dissipation losses associated with each material. The Kapton substrate was assigned a relative permittivity of 3.2 and loss tangent of 0.009; these values were obtained from the manufacturer’s specification sheet. The conductivity of the CPW signal line was varied from that of bulk silver to those measured for the conductive inks to investigate ohmic loss in the cases studied.

A Rohde & Schwarz ZVB20 (Columbia, MD, USA) vector network analyzer (VNA) was used to measure the scattering parameters of the fabricated CPW samples over the 1–12 GHz frequency range. DuPont Kapton HN polyimide (Wilmington, DE, USA) [20] with a thickness of 125 µm and dielectric constant of ~3.2 at 1 GHz served as the substrates for the CPWs. Two SMA connectors were attached using soldered indium to the two ends of each CPW line to facilitate RF characterization. Thermally evaporated Ag that was deposited using 99.99% purity pellets from Kurt J. Lesker (Jefferson Hills, PA, USA), a base pressure of ~10^−6^ Torr, and a rate of 1 nm/sec served as the control for benchmarking. The thickness was 2.5 μm for all CPW samples, which is 3.9 times the skin depth at 10 GHz for fully dense Ag. A laser-machined shadow mask defined the CPW dimensions with the only difference between the control and the printed samples being that the signal line was evaporated in the control but printed otherwise.

As previously mentioned, a VNA (Rohde & Schwarz ZVB20) was used to measure the scattering parameters of the fabricated CPW samples over the 1–12 GHz frequency range. Prior to conducting measurements, a Rohde & Schwarz ZN-Z52 (Columbia, MD, USA) electronic calibration kit was used to correct for the loss of the test cables connected to the two VNA ports. The calibration process also put the reference planes (for phase measurement) at the input/output of the two SMA connectors at the two ends of the CPW lines. The total loss of the fabricated CPW line as a function of frequency was calculated from the measured values of the input reflection coefficient (S_11_) and the transmission coefficient (S_21_). In general, this total loss is a combination of several factors, including the ohmic and dielectric losses of the SMA connectors, the dielectric loss of the Kapton substrate, ohmic loss of the metallic layer used to implement the CPW, and radiation leakage of the line.

## 3. Results and Discussion

### 3.1. Electrical Resistivity

Table 1 summarizes the DC resistivity of both inks in their as-printed and post-thermal exposure conditions as described in the experiment where each reported resistivity value is an average taken from at least 3 prints. It is observed that at 300–800 μΩ·cm, the resistivity of the as-printed NF suspension was significantly larger than the 21.4 μΩ·cm of the NPR ink, which could be ascribed to its polymeric binder. After being subjected to the manufacturer’s curing routine, the resistivity of the NF ink was 3.7 μΩ·cm, and the corresponding value for the NPR formulation was 7.9 μΩ·cm. Although these values are ~2× and 5× larger than the resistivity of bulk Ag (1.6 μΩ·cm), they nonetheless indicate that an interconnected conductive network was established. After 225 °C for 30 min, the resistivity of the NF ink decreased to 2.1× the bulk value, which improved to 1.4× after being exposed to 350 °C for the same time. The corresponding values after equivalent treatments of the NPR Ag ink were 3.7× and 2.2×, respectively.

### 3.2. Thermally Driven Microstructural and Chemical Evolution

**Figure 1:**
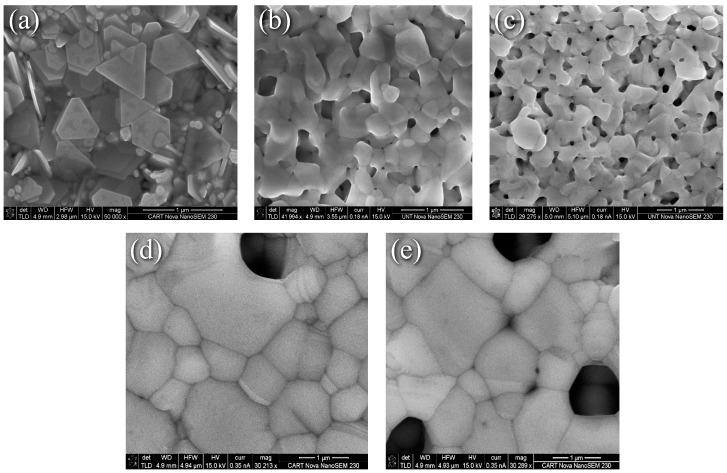
SEM images of nanoflake ink (**a**) As-printed, (**b**) 225 °C for 5 min, (**c**) 225 °C for 30 min, (**d**) and (**e**) 350 °C for 30 min.

Representative SEM images of nanoflake specimens and their surface evolution with heat treatments are summarized in Figure 1. The as-printed surface is a partially stacked assortment of platelets with well-defined edges and corners. Where the edges meet can be easily discerned from Figure 1a, and binder coated flake surfaces and edges are visible throughout. Figure 1b revealed that heating at 225 °C for 5 min as recommended by the manufacturer eliminated the binder and produced a surface morphology that can be characterized as the intermediate stage of sintering [21], where extensive necking, coalescence, and an interconnected pore structure are obtained. Diffusion driven by the reduction in surface energy results in the corners becoming rounded, and the sharp edges becoming distorted. In addition, edge-to-edge, corner-to-corner, and corner-to-edge connections can be discerned. All of these microstructural features enhance diffusion, increase density, and provide more connections for electrical transport. In addition, fusion is observed between the faces of flakes lying on top of others, which further enhances diffusion and electrical transport pathways across the thickness of the print. Increasing the dwell time to 30 min at 225 °C resulted in the interconnected pore structure becoming more defined and isolated pores emerging as Figure 1c shows. The mechanism described by Wang et al. is likely applicable [22] in which necking predominantly begins at the tips where the diffusion potential gradient is largest, then progresses to edges and faces. Heating to 350 °C for 30 min resulted in significant grain growth, pore closure, and densification as Figure 1d,e reveals, although all interconnected pores were not eliminated. This suggests an incomplete, near-final stage of sintering [21]. Thermal grooving [23] and twinning are also observed at this temperature, and it can be also seen that the angle between grains is not the same throughout, which is possibly due to the dependence of grain boundary energy on grain misorientation, adsorbed impurities from the binder on the surface, or the variation of Ag-ambient air interface energy with crystal orientation.

**Figure 2:**
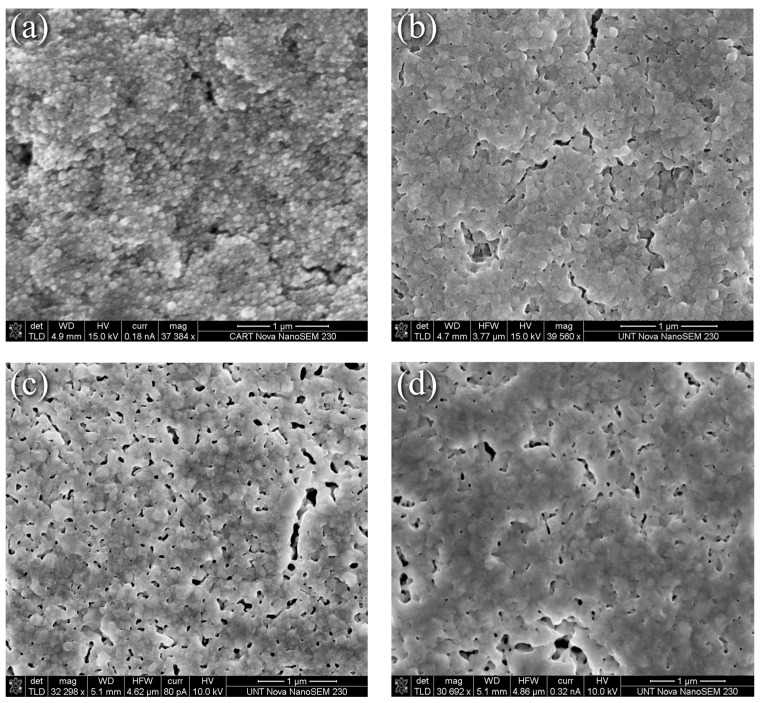
SEM images of nanoparticle/reactive ink (**a**) As-printed, (**b**) 100 °C for 20 min, (**c**) 225 °C for 30 min, (**d**) 350 °C for 30 min.

In contrast to the evolution seen in Figure 1, the thermally induced changes to the nanoparticle-reactive formulation were less dramatic as the representative SEM surface morphology images in Figure 2 depict. Here, Figure 2a through Figure 2d correspond to the nanoparticle-reactive ink in its as-printed condition, after curing per the manufacturer’s recommendation at 100 °C for 20 min, and after heating at 225 °C and 350 °C for 30 min, respectively. Figure 2a shows that the as-printed specimens are characterized by individual and agglomerated nanoparticles that are held together and coated by a combination of precipitated Ag and the remaining reactive phase. Heating for 20 min at 100 °C as recommended by the manufacturer produced necking and increased interparticle contact as Figure 2b shows, where a porous interconnected matrix that envelopes the nanoparticles forms. The MOD process generates new, smaller nanoparticles, thus creating a distribution of Ag particles in an Ag “matrix”. Upon heating to 225 °C for 30 min, the interconnected pore structure grew and became more defined as seen in Figure 2c. This condition, for the most part, persists at 350 °C for 30 min where fewer but larger pores are visible. The clear coarsening and pore elimination, i.e., densification, seen in Figure 1d,e for the nanoflake ink were not obtained in the NPR case. Further discussion with the benefit of XRD grain size and XPS analyses are presented in the sections that follow.

**Figure 3:**
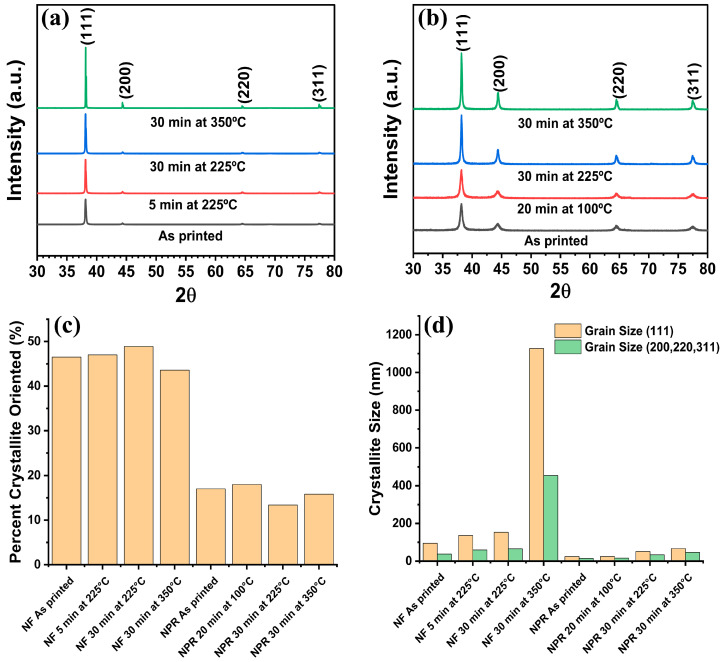
XRD analysis. (**a**) Nanoflake ink, (**b**) Nanoparticle-reactive ink, (**c**) Percentage of (111) oriented crystallites, (**d**) Crystallite size from Rietveld refinement.

Figure 3 shows the XRD spectra of the subject samples from which the grain size data in Table 2 were obtained. Comparison of Figure 3a with Figure 3b reveals that all samples exhibited (111) texture with (111) planes defined as planes parallel to the substrate, but the NF ink showed greater texture than the NPR ink. The percent (111) orientation obtained from the March–Dollase parameter was 44–49% and 13–18% for the NF and NPR samples, respectively. No additional phases other than Ag were observed. The crystallite size distribution calculated using Rietveld refinement is presented in Figure 3d, where NF refers to the nanoflake ink and NPR indicates the nanoparticle-reactive formulation. These data show that modest crystallite growth is obtained by heating the NF ink at 225 °C for 30 min rather than the manufacturer-recommended 5 min and that heating at 350 °C for 30 min produced significantly enlarged grains. Considering the (111) orientation specifically, the ~95 nm as-printed crystallites grew to 136 nm when cured per the manufacturer’s recommendations at 225 °C for 5 min, increased to ~153 nm after holding the same temperature for 30 min, and were significantly larger at 1128 nm after 30 min at 350 °C.

In the NPR case, the as-printed crystallites were ~24 nm and remained essentially unchanged at ~25 nm after using the recommended 100 °C for 20 min. Heating at 225 °C for 30 min doubled the size to ~51 nm, which increased to only ~67 nm at 350 °C for 30 min. It is theorized that byproducts from curing the reactive phase and other chemical impurities from the MOD reaction remain significant at 100 °C for 20 min. These impurities segregate to the grain boundaries and pore surfaces where they impede diffusion, the mechanism by which the grains grow. Thus, the grain size is pinned at ~25 nm [24]. At higher temperatures, impurities mostly desorb or otherwise volatilize, and diffusion and modest crystallite growth is restored. These data indicate that the influence of the reactive phase is significant, and care is warranted when applying descriptions of nanoparticle-only sintering (e.g., [25,26]).

A review of the resistivity data in Table 1 in conjunction with the crystallite sizes in Table 2 reveals that treatment at 350 °C for 30 min yielded the lowest resistivity and largest grain size for both ink formulations. The obtained electrical resistivities of the printed NF and NPR Ag were 1.4× and 2.2× larger than bulk Ag, respectively. Compared to each other, the NF was 1.6× smaller. However, the corresponding grain sizes were 1128 nm and 67 nm, a factor of ~16.8× between them. The data show that after 30 min at 225 °C the NF grain size and resistivity were 135 nm and 3.4 μΩ·cm, respectively. After 30 min and a higher temperature of 350 °C, the NPR grain size was only 66.9 nm, whereas the resistivity was essentially *the same* at 3.5 μΩ·cm. It should be noted that the electron mean free path in Ag is 53.3 nm [27], which is in the same order or *smaller* than the crystallite sizes obtained at 225 and 350 °C. Thus, the data indicate that while direct current resistivity decreases with increasing grain size, beyond some dimension, grain boundary scattering is no longer the determinant preventing bulk electrical transport.

**Figure 4:**
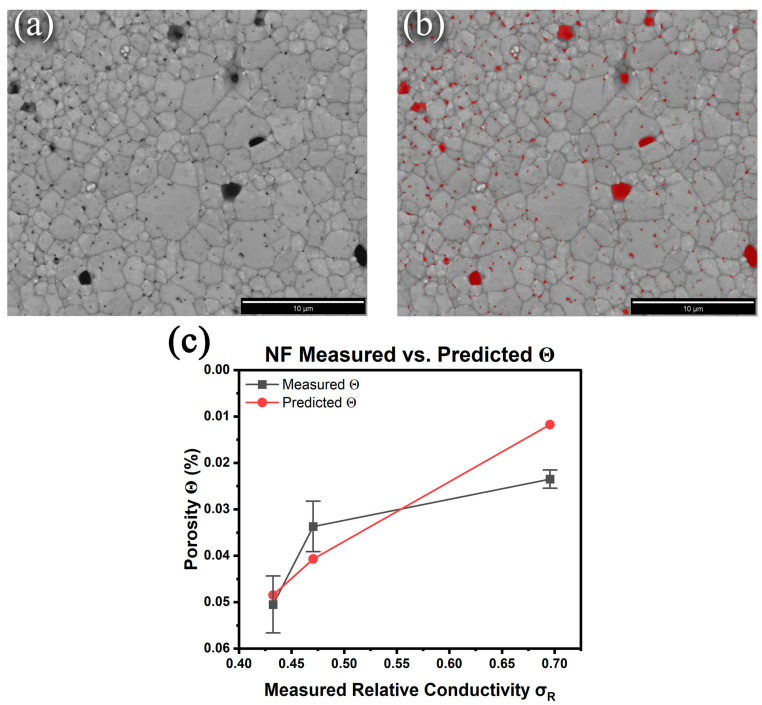
Nanoflake ink 350 °C 30 min BSE SEM image (**a**) before and (**b**) after MIPAR porosity analysis. (**c**) Comparison of the measured porosity and predicted porosity using Equation (3).

Given the inadequacies of the Archimedes or pycnometry methods for determining the density/porosity of micron-sized prints with nanometer-scale pores, the suitability of digital imaging together with the general equations proposed by Cuevas et al. [28] for correlating conductivity with porosity was assessed. These researchers showed that based on percolation theory, the following equation produced excellent fits:

(3) $$\sigma_{R}={\left(1-\Theta^{s}\right)}^{t}$$

where $\sigma_{R}$ is the relative conductivity (quotient of the measured to bulk conductivity), Θ is the % porosity (the relative density is (1−Θ)), and *s* and *t* are treated as fitting parameters that typically range from 0.4 to 1.4 and 1 to 4, respectively. To estimate the porosity of the sintered samples, high-resolution backscattered electron images (BSE) of the top surfaces were collected, and the visible pores were identified using MIPAR image analysis software to calculate the % porosity [Figure 4b]. Here, the approximation is that the top surface is representative of the bulk pore structure. Six images from random locations across the samples were collected, and the porosity measurements from the MIPAR analysis are summarized in Table 3. The model (Equation (1)) was fit to the experimentally measured NF relative conductivities to predict their porosities, generating s and t values of 0.550 and 4.00, respectively, with a determination coefficient R^2^ of 0.999 [Figure 4c]. The data show that a model to predict the relative porosity of sintered DIW prints based on their measured conductivities is feasible. However, it should be noted that the error in the MIPAR-determined porosity is unknown as it only analyzes the surfaces of the prints as acknowledged earlier. For example, closed porosity within the samples that remain unaccounted for cannot be ruled out. Furthermore, the model proposed by Cuevas et al. [28] does not account for remnant chemical impurities within the prints that also contribute to decreased conductivity. Nonetheless, the preliminary determinations are encouraging; however, a dedicated study with a full set of porosities collected from multiple ink chemistries and thermal treatments will be necessary to fully validate the model for predicting conductivity from porosity and vice versa.

**Figure 5:**
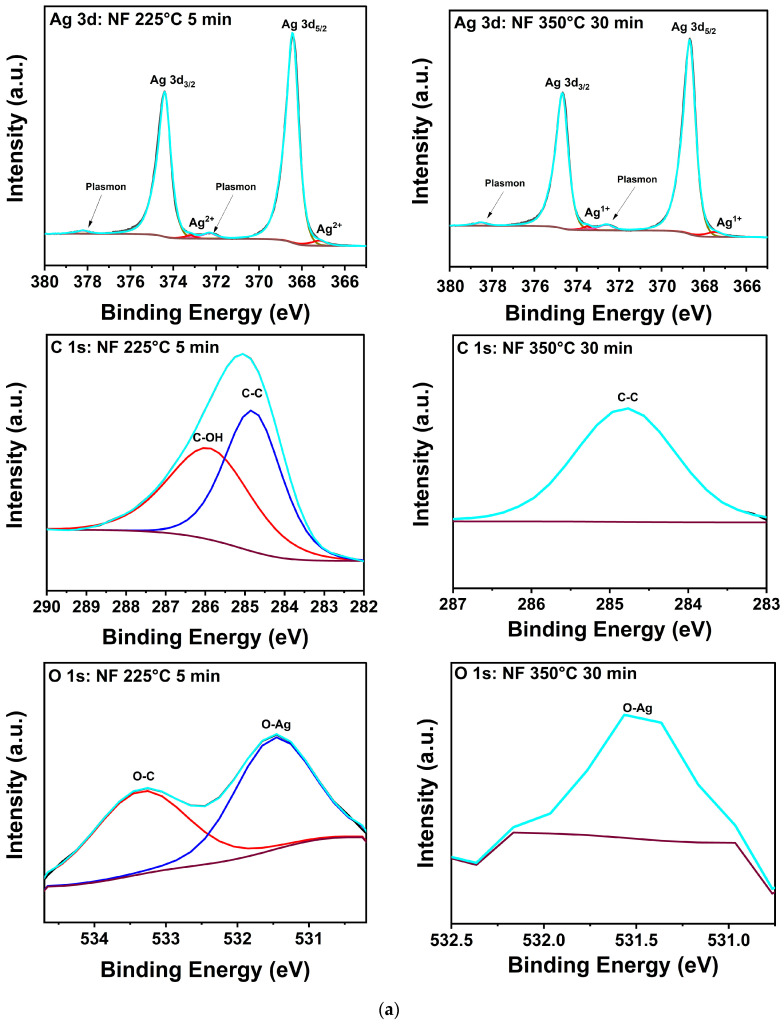

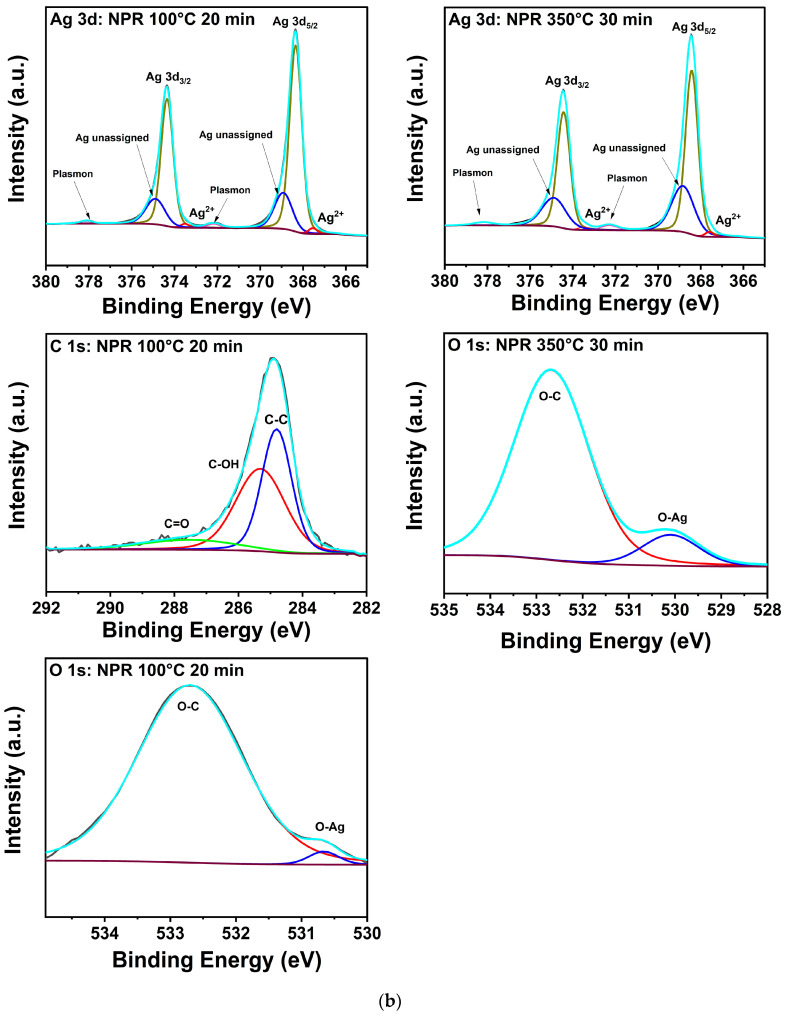
(**a**). XPS composition of printed Ag nanoflake ink after (**left**) curing for 20 min at 100 °C and (**right**) 30 min at 350 °C. (**b**) XPS composition of printed Ag nanoparticle-reactive ink after (**left**) curing for 20 min at 100 °C and (**right**) 30 min at 350 °C. The adjacent labels correspond to their respective colored lines (deconvoluted peaks). The grey line corresponds to the raw data. The dark purple line corresponds to the background. The cyan line corresponds to the fitted data (envelope).

The salient features of the NF XPS spectra for the manufacturer’s and 350 °C conditions presented in Figure 5a are qualitatively representative in that only Ag, O-Ag, O-C, C-O, or C-C bonding [29,30,31,32,33,34,35,36,37] were detected in all samples. The peak positions and assignments for both formulations are summarized in Table 4, and the atomic percentages of Ag, C, and O from the samples are presented in Table 5. The latter reveals that all treatments of the NF prints yielded an increase in Ag at% accompanied by a decrease in C and O at%. For the NF sample treated at 350 °C for 30 min, trace amounts of C and O were present at 0.4 and 0.2 at%, respectively, denoting effective decomposition and desorption of the binder and any Ag oxides or sub-oxides. The C-C bonding and weak O signal measured at 350 °C indicate that these findings are likely due to adventitious contamination. The shoulders observed at 367.2 and 373.2 eV in the spectra of NF Ag treated at 225 °C for 5 min are assigned to Ag^2+^ in AgO. In the samples subjected to 350 °C for 30 min, these peaks shifted to 365.5 and 373.4 eV, respectively, consistent with the decomposition of AgO to Ag_2_O [33,34]. In a previous study by Gao et al. [38], it was recorded that the thresholds for thermal decomposition of AgO and Ag_2_O were 200 and 300 °C, respectively, with the Ag_2_O reaction being reversible. However, other studies show that AgO decomposition into Ag_2_O and O begins at 160 °C and Ag_2_O transforms into Ag and O at about 380 °C [39]. However, other results indicate that the transformation of AgO to Ag_2_O occurs in the 200 to 300 °C range, and that Ag_2_O is stable up to 400 °C [40]. In summary, while the specific reported transformation temperatures vary, there is consensus for the following reactions: 2AgO → Ag_2_O +1/2O_2_, Ag_2_O → 2Ag + 1/2O_2_.

The corresponding spectra for the NPR ink in Figure 5b and its composition data show more complexity likely due to contributions from the reactive component. As Table 5 shows, C-C bonding peaked at 100 °C but fell below the detection limit of the spectrometer at higher processing temperatures; the C measured at 100 °C was no longer detected at the higher processing temperatures. These data suggest that remnant C is driven to the surface at 100 °C where it mostly desorbs at 225 and 350 °C, but some of it is also sequestered by bonding to O. On the other hand, oxygen, which decreased to a noisy 0.2 at% signal at 350 °C for the NF samples, steadily increased to a significant ~8.3% in the NPR case as Table 5 reveals. Furthermore, in addition to AgO bonding, unidentified Ag peaks are observed at 368.9 and 374.9 eV, and it is noteworthy that they remained after treatment at 350 °C for 30 min. Integration of the area under the relevant curves reveals that Ag in the unidentified environment increased from ~22% to 36% with 350 °C heat treatment versus the manufacturer’s recommendation. The corresponding analysis for O shows that under the manufacturer’s recommended condition, ~98% is bonded to C and 2% to Ag. At 350 °C, ~89% of it is bonded to C and ~11% to Ag. Because of the high porosity and small grain structure of the NPR samples, ample surface area is available for trapping binder residuals and reactions with oxygen as indicated by the data obtained with increasing treatment temperature. Thus, it is proposed that the unidentified Ag peaks are likely due to Ag bonded to O in a mixture of Ag_2_O, Ag_x_O, and remnants of the metal-organic precursors [41]. This contamination that decorates pore surfaces and grain boundaries likely reduces the driving forces for diffusion, densification, and grain growth that were qualitatively recorded in the SEM and XRD results for the NPR samples.

### 3.3. RF Performance after Heat Treatment

**Figure 6:**
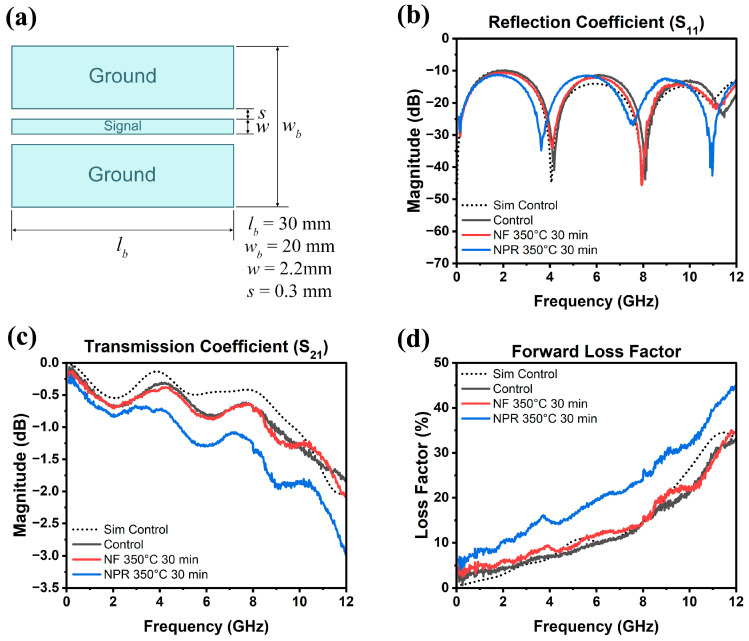
(**a**) CPW dimensions and measured (**b**) reflection coefficient, (**c**) transmission coefficient, (**d**) forward loss factor as a function of frequency up to 12 GHz. The dotted curve is the simulation of the evaporated Ag control.

**Figure 7:**
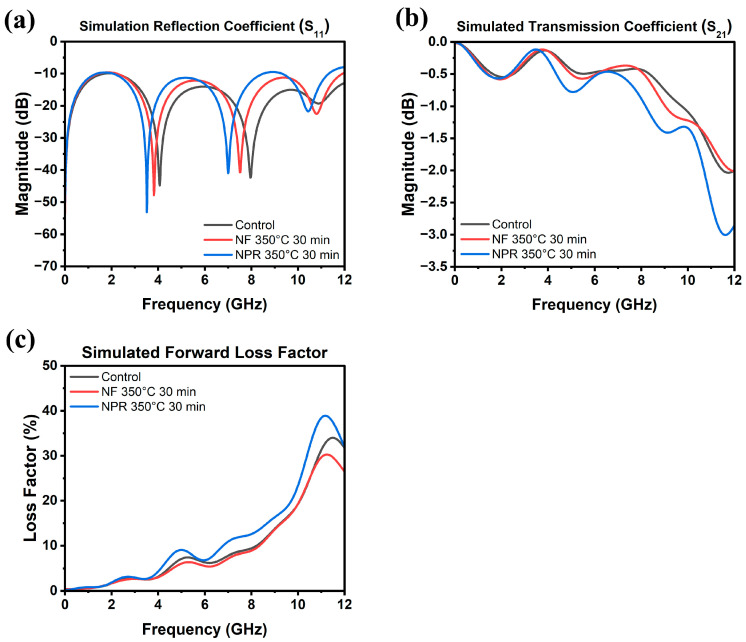
Simulated (**a**) reflection coefficient, (**b**) transmission coefficient, and (**c**) forward loss factor as a function of frequency up to 12 GHz.

The results of the RF measurements are presented in Figure 6, where Figure 6a is a schematic of the CPW showing its dimensions. Full-wave simulation data for the three samples are shown in Figure 7 for reference. As Figure 6b shows, the S_11_ reflection coefficients of the NF and NPR samples were similar to that of the thermally evaporated Ag control over the measured frequency range. The trend in the reflection coefficients of the three cases agrees well with what is observed from the simulation results shown in Figure 7a. However, the transmission coefficient data in Figure 6c reveal that while the S_21_ values are comparable between the control and NF samples, the corresponding values for the NPR equivalent are noticeably smaller. Converting dB to the linear scale, the transmission coefficients for the NF prints are 0.87 and 0.75 at 5 GHz and 10 GHz, respectively. The corresponding values for the NPR specimens are 0.75 and 0.65, respectively. As a result, the NPR formulation also exhibited a larger forward loss factor ($FLF=1-{\left|S_{11}\right|}^{2}-{\left|S_{21}\right|}^{2}$) [42] as the comparison in Figure 6d illustrates. The simulation results shown in Figure 7b,c also confirm that the performances of the control and NF samples are very close to each other in terms of the transmission coefficient and forward loss factor. While the simulations predicted that the NPR sample would have the highest loss among the three cases, the measured S_21_ of this sample is larger than predicted. It should be noted that some discrepancies between numerical and experimental results are expected due to factors not taken into account in the simulation models. For example, the actual SMA connectors that were used may be more lossy than the simulated connectors despite our best attempts at creating a representative model. Additionally, soldering of the SMA connectors to the CPW was accomplished using indium, which was omitted in the numerical model for simplification. Further, a small air gap (e.g., on the order of a few tenths of a mm) between the flange of the SMA connectors and the edge of the Kapton substrate was unavoidable in many cases despite using a mechanical fixture to hold the components in place to maintain consistency when soldering. Numerical investigations showed that the S_21_ value of the CPW line is quite sensitive to small variations of this air gap. Moreover, any uncertainty in the loss tangent of the Kapton film would move the S_21_ curve up or down. Nevertheless, both simulation and measurement results demonstrate higher S_21_ losses of the NPR sample compared to the NF and control cases. The higher ohmic losses in the NPR sample are associated with its porosity, chemical impurities, and microstructure that were revealed from the detailed SEM, XPS, and XRD analyses. More detailed studies to understand the grain boundary and pore structure and chemistries are necessary since differences with the grain interiors represent dielectric constant mismatches, dissimilar carrier relaxation times, and potential trapping that may underpin losses, particularly at a high frequency.

## 4. Conclusions

In summary, a comparative analysis of the microstructures, chemistries, and electrical responses of thermally treated Ag nanoflake suspension and nanoparticle reactive inks show that the achievable RF performance in printed Ag transmission lines is comparable to that of thermally evaporated Ag when thermal treatments produce a chemical purity, density, and grain size that are close to those of the bulk material. Otherwise, remnant chemical impurities at grain boundaries and the surface of pores negatively affect diffusion, resulting in small grain sizes and poor densification. Further, impurities introduce interfacial trapping and associated relaxation times that degrade frequency response. Therefore, a co-design approach should be utilized. The ink formulation at the outset must consider the required electrical performance and the thermally driven processes that govern the final microstructure and chemical purity of prints: desorption of the dispersing phase, grain growth, porosity elimination, and densification. The success of these processes in turn depends on the chemical composition, particle size and shape, and other driving forces for diffusion that the ink design must facilitate.

## Figures and Tables

**Table 1:** Summary of dc resistivities of the two inks.

Nanoflake Suspension (NF)		
Treatment	Resistivity (μΩ·cm)	Multiple of bulk Ag (ρ=1.6 μΩ·cm)
As printed	300–800	
Cured per manufacturer’s recommendations for 5 min at 225 °C	3.7 (±0.1)	2.3×
30 min at 225 °C	3.4 (±0.3)	2.1×
30 min at 350 °C	2.3 (±0.1)	1.4×
**Nanoparticle-reactive (NPR)**		
As printed	21.4 (±2.6)	
Cured per manufacturer’s recommendations for 20 min at 100 °C	7.9 (±0.3)	4.9×
30 min at 225 °C	5.7 (±0.2)	3.7×
30 min at 350 °C	3.5 (±0.3)	2.2×

**Table 2:** Summary of crystallite size and percentage (111) orientation for the two inks.

Nanoflake Suspension (NF)			
Treatment	Grain size (111) (nm)	Grain size (111, 220, 311) (nm)	Percent (111) oriented (%)
As printed	95.4	37.5	46.5
Cured per manufacturer’s recommendation for 5 min at 225 °C	135.6	59.4	47
30 min at 225 °C	135	65	48.9
30 min at 350 °C	1127.7	453.9	43.6
**Nanoparticle-reactive (NPR)**			
As printed	24.4	14.5	17
Cured per manufacturer’s recommendation for 20 min at 100 °C	24.7	15.7	18
30 min at 225 °C	51.2	33.9	13.4
30 min at 350 °C	66.9	46.8	15.8

**Table 3:** Summary of porosity measurements from MIPAR.

Ink	Heat Treatment	% Porosity
NF	Cured per manufacturer’s recommendation for 5 min at 225 °C	5.05 ± 0.61
NF	30 min at 225 °C	3.37 ± 0.54
NF	30 min at 350 °C	2.35 ± 0.19

**Table 4:** XPS peak assignment [29,30,31,32,33,34,35,36,37].

NF	Peak Position (eV)	Notes
Ag 3d_5/2_	368.4	Metal, plasmon at 372.3 eV
Ag 3d_3/2_	374.4	Metal, plasmon at 378.2 eV
Ag^2+^	367.2	AgO
Ag^2+^	373.2	AgO
Ag^1+^	367.5	Ag_2_O
Ag^1+^	373.4	Ag_2_O
C-O	285.9	
C-C	284.8	
O-C	532.8	
O-Ag	531.1	
**NPR** (in addition to the above)		
Unassigned Ag	368.9 and 374.9	Ag in remnants of the metal-organic precursors
C=O	287.5	

**Table 5:** Summary of composition from XPS.

Nanoflake Suspension (NF)			
Treatment	Atomic % Ag	Atomic % C	Atomic % O
As printed	74.9	23.9	1.2
Cured per manufacturer’s recommendation for 5 min at 225 °C	88.9	10.7	0.4
30 min at 225 °C	97.5	2.0	0.5
30 min at 350 °C	99.4	0.4	0.2
**Nanoparticle-reactive (NPR)**			
As printed	86.5	10.8	2.7
Cured per manufacturer’s recommendation for 20 min at 100 °C	74.7	22.8	2.5
30 min at 225 °C	95.5	undetectable	5.5
30 min at 350 °C	91.7	undetectable	8.3

## Data Availability

Data are contained within the article.
